# Tandem Visual Recognition of Cu^2+^ and Chiral Tartaric Acid by Sequence Gel Formation and Collapse

**DOI:** 10.3390/gels11050340

**Published:** 2025-05-01

**Authors:** Jian Zeng, Yixuan Jiang, Xiao-Qi Yu, Shanshan Yu

**Affiliations:** Key Laboratory of Green Chemistry and Technology, Ministry of Education, College of Chemistry, Sichuan University, Chengdu 610064, China; zj_zengjian@163.com (J.Z.); j_yixuan@163.com (Y.J.); xqyu@scu.edu.cn (X.-Q.Y.)

**Keywords:** visual sensor, metal–organic gel, chiral recognition

## Abstract

A chiral gelator (*R*)-H_6_L with multiple carboxyl groups based on a 1,1′-bi-2,2′-naphthol (BINOL) skeleton was prepared, and it could form a supramolecular gel under the induction of water in DMSO/H_2_O and DMF/H_2_O (1/1, *v*/*v*). In the EtOH/H_2_O system, the original partial gel transformed into a stable metal–organic gel (MOG), specifically with Cu^2+^ among 20 metal ions. It is proposed that Cu^2+^ coordinates with the carboxyl groups of (*R*)-H_6_L to form a three-dimensional network structure. With the addition of a variety of α-hydroxy acids and amino acids, the Cu^2+^-MOG collapsed with merely 0.06 equivalents of L-tartaric acid (L-TA), while other acids required much larger amounts to achieve the same effect, realizing the visual chemoselective and enantioselective recognition of tartaric acid. Therefore, the chiral gelator (*R*)-H_6_L achieved the tandem visual recognition of Cu^2+^ and chiral tartaric acid by sequence gel formation and collapse, offering valuable insights for visual sensing applications and serving as a promising model for future chiral sensor design.

## 1. Introduction

Molecular detection is mainly achieved through specific interactions between a sensor molecule and its substrates [1], and the recognition process has been extensively analyzed using modern high-precision instrumental analytical techniques, including UV [2], fluorescence [3], circular dichroism (CD) [4], nuclear magnetic resonance (NMR) [5], high-performance liquid chromatography (HPLC) [6], etc., which require expensive instruments and specialized operation. In comparison, visual detection methods, such as color change methods [7], precipitation formation [8], gel formation [9], and collapsing [10], are much more convenient and direct.

As stimuli-responsive materials, supramolecular gels have been used for recognition by color change or gel–solution transformation through non-covalent interactions, including π–π stacking, hydrogen-bonding interactions, metal–ligand interactions, ion interactions, and van der Waals interactions [11,12,13,14,15,16,17]. For example, Li and co-workers prepared a quadruple-stimuli-response Eu(III)-MOG, which recognized K^+^ through color changes based on host–guest interactions [18]. Pu and co-workers reported a chiral Cu^2+^-MOG gel, which achieved the enantioselective recognition of phenylglycine through the displacement reaction with Cu^2+^ [19]. Zhang and co-workers prepared histidine-derived amphiphiles, which showed the capacity for selective gelation towards tartaric acid enantiomers [20]. At present, visual recognition through gels is limited by the substrate type and sensitivity, and it is mostly achieved through only the formation or collapse of a gel. Therefore, a sensitive tandem visual recognition method based on sequential gel formation and collapse is proposed.

Herein, a chiral compound, (*R*)-H_6_L, was designed and synthesized, featuring an axial chiral BINOL core for chiral induction and four carboxylic acid groups for hydrogen-bonding interaction. The addition of Cu^2+^ improved the gelation ability, and it formed a (*R*)-H_6_L-Cu^2+^ gel in EtOH/H_2_O (1:1, *v*/*v*) to realize the specific recognition of Cu(II) among 20 metal salts. Meanwhile, the (*R*)-H_6_L-Cu^2+^ gel exhibited highly sensitive visual chemoselective and enantioselective collapse toward L-tartaric acid (L-TA), even at ultralow concentrations (as low as 0.06 equiv.), compared to enantiomers of α-hydroxy acids and amino acids.

## 2. Results and Discussion

### 2.1. Synthesis and Gelation Ability of (R)-H_6_L

**Scheme 1:**
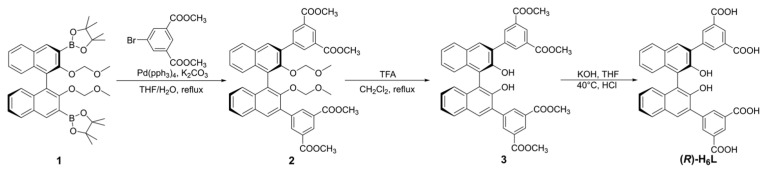
Synthesis of (*R*)-H_6_L.

The gelator (*R*)-H_6_L was synthesized (Scheme 1 and Figures S1–S5). In our previous work, the synthesized (*R*)-H_3_L-Cu^2+^ metal–organic gel was able to achieve the sensitive visual chemoselective and enantioselective recognition of histidine [21]. After increasing the carboxyl groups and hydroxyl groups and combining chiral binaphthol as the skeleton, (*R*)-H_6_L was expected to form enhanced hydrogen-bonding interactions, which contributed to strengthening its gelation abilities and further improving the selectivity and sensitivity of recognition.

The gelation properties of compound (*R*)-H_6_L in various solvents were systematically evaluated using the inverted vial method. As shown in Table 1, (*R*)-H_6_L exhibited good solubility in polar organic solvents, including acetone, THF, DMF, DMSO, 1,4-dioxane, i-PrOH, EtOH, and MeOH. The high solubility in these solvents comes from its multiple carboxyl groups, which engage in hydrogen bonding with polar solvents. The addition of water to organic solutions of (*R*)-H_6_L may induce the formation of gels; therefore, mixed-solvent systems of these organic solvents and water were screened. It was found that both the type of organic solvent and the fraction of water affected the gelation process of (*R*)-H_6_L. As shown in Figure S6, the stable gel formation of (*R*)-H_6_L in DMSO was induced exclusively under conditions containing 50% water by volume at equivalent concentrations. Deviations from this optimal ratio resulted in distinct phase behaviors: systems with <50% water remained as homogeneous solutions, while those with >50% water led to sol formation or precipitation. Among them, only in mixed solvents of DMF/H_2_O (1/1, *v*/*v*) and DMSO/H_2_O (1/1, *v*/*v*) did it form a stable gel, with a critical gelation concentration (CGC) at 20.3 mM and 14.8 mM, respectively. The lower CGC in the DMSO/H₂O system may be attributed to DMSO’s stronger hydrogen-bond-accepting capacity compared to DMF, which enhances the thermodynamic stability of the gel network. A partial gel was formed in EtOH/H_2_O and MeOH/H_2_O. For the remaining four solvents, the addition of water caused no change to the (*R*)-H_6_L solution. All the studied gels were formed by heating to completely dissolve them followed by cooling to room temperature, and they were judged using the inverted vial method.

### 2.2. Specific Recognition of Cu^2+^ by Gel Formation

**Figure 1:**
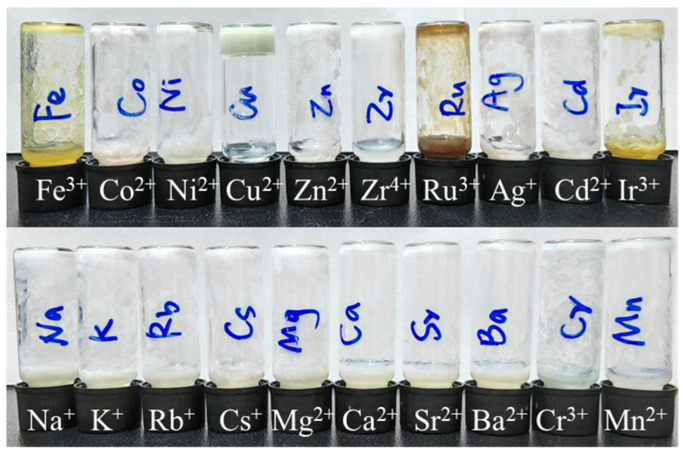
The gelation of (*R*)-H_6_L (14.8 mmol) coordinated with 2 equiv. of various metal ions in EtOH/H_2_O (1/1, *v*/*v*).

Motivated by the pronounced metal-coordinating affinity of carboxyl groups, we then investigated the metal ion responses of (*R*)-H_6_L through its gelation behavior. All metal ions were employed as their chloride salts to eliminate interference from anions (except AgNO_3_). The effects of metal ions on the gelation ability of (*R*)-H_6_L were first studied in EtOH/H_2_O, since only a partial gel was formed in this solvent. When 2 equiv. of 20 common metal salts were added to the partial gel of (*R*)-H_6_L (14.8 mmol in EtOH/H_2_O 1/1, *v*/*v*), it was found that only Cu^2+^ induced the formation of a stable light green gel, while the other metal salts caused either a partial gel or precipitation (Figure 1). The CGC was determined to be 4.5 mM. The other seven solvent systems mentioned above (organic solvent/H_2_O, 1/1, *v*/*v*) were also tested using 14.8 mM (*R*)-H_6_L with 2 equiv. of Cu^2+^ (Figure S7). The stable gel remained and was strengthened in DMF/H_2_O and DMSO/H_2_O, and the CGC in DMSO/H_2_O was significantly reduced from 14.8 mM to 1.8 mM. For the remaining solvent systems, it appeared as a solution or precipitation occurred with no gel formation. These results demonstrate that (*R*)-H_6_L achieved the visual specific recognition of Cu^2+^ in EtOH/H_2_O (1/1, *v*/*v*) through gel formation, which is ascribed to the strong metal coordination of carboxylic acid groups with Cu^2+^, enhancing the stability of the gel and the fixation ability of the solvent. The gelation response of (*R*)-H_6_L to cuprous ions (Cu⁺) was also examined. Copper(I) halides are poorly soluble in ethanol and water; therefore, we modified the testing protocol by adding the solvent to a fixed amount of solid. Under these conditions, a turbid solution formed without gelation.

**Figure 2:**
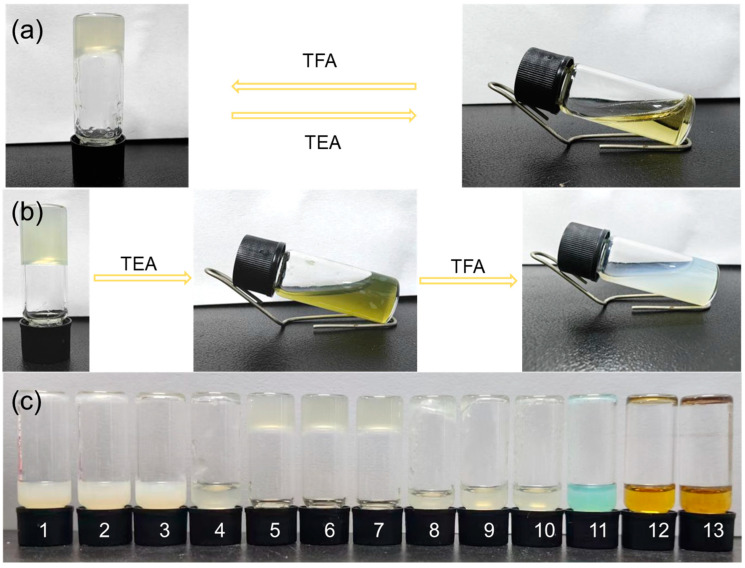
The pH-stimuli response of (**a**) (*R*)-H_6_L (14.8 mM) in DMSO/H_2_O (1/1, *v*/*v*) and (**b**) the MOG (*R*)-H_6_L-Cu^2+^ (4.5 mM with 2 equiv. of Cu^2+^) in EtOH/H_2_O (1/1, *v*/*v*). (**c**) The gelation of (*R*)-H_6_L (4.5 mM in EtOH/H_2_O (1/1, *v*/*v*)) with 2 equiv. of Cu^2+^ at different pH levels.

The pH-stimuli responses of the supramolecular gel (*R*)-H_6_L and the MOG (*R*)-H_6_L-Cu^2+^ were tested. As shown in Figure 2a, the addition of 20 μL triethylamine (TEA) to the (*R*)-H_6_L gel (14.8 mM) in DMSO/H_2_O (1/1, *v*/*v*) resulted in a gel–sol transition, while the gel reformed after an equal amount of trifluoroacetic acid (TFA) was subsequently added to the above solution. However, the MOG (*R*)-H_6_L-Cu^2+^ (4.5 mM) in EtOH/H_2_O (1/1, *v*/*v*) did not exhibit this reversible response (Figure 2b). The addition of 20 μL TEA transformed the gel into an olive-colored solution, and then it precipitated and formed a white suspension after the addition of 20 μL TFA. The difference between them was based on their main interactions: hydrogen bonding in supramolecular gels and metal–ligand coordination bonds in metal–organic gels. The continuous addition of alkalis and acids did not change the pH and hardly affected the hydrogen bonding. For the MOG (*R*)-H_6_L-Cu^2+^, TEA competed with (*R*)-H_6_L to coordinate with Cu^2+^ and thus disrupted the gel. Following this, a systematic investigation was conducted on the gelation ability of (*R*)-H_6_L (4.5 mM) in EtOH/H_2_O (1/1, *v*/*v*) and Cu^2+^ in different pH environments. As shown in Figure 2c, gel formation occurred only under neutral or weakly acidic conditions. Under either acidic or alkaline conditions, the formation of gels was notably hindered. When the pH was below 3, the solution exhibited a white turbid appearance, attributed to H⁺ interfering with the hydrogen-bonding interactions between (*R*)-H_6_L and the solvent molecules, thereby decreasing its solubility. It was unable to form a gel at a pH exceeding 8. Concurrently, the solution exhibited progressive color intensification with rising hydroxide ion (OH⁻) concentrations, which was attributed to the formation of copper(II)-based coordination complexes.

**Figure 3:**
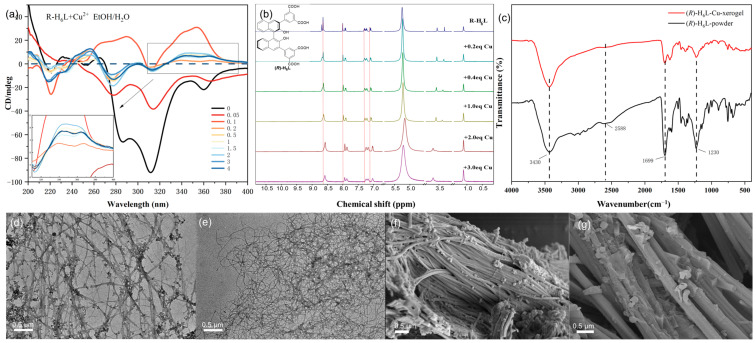
(**a**) CD spectra of (*R*)-H_6_L (4.5 mM) + Cu^2+^ with different equivalents in EtOH/H_2_O (1/1, *v*/*v*); (**b**) ^1^H NMR spectra (EtOD-*d*_6_) of (*R*)-H_6_L with different equivalents of Cu^2+^; (**c**) FT-IR spectra of (*R*)-H_6_L powder and (*R*)-H_6_L-Cu_2_ xerogel; TEM image of dilute (**d**) (*R*)-H_6_L-Cu_0.2_ gel and (**e**) (*R*)-H_6_L-Cu_2_ gel obtained from EtOH/H_2_O (1/1, *v*/*v*); SEM image of (**f**) (*R*)-H_6_L-Cu_0.2_ xerogel and (**g**) (*R*)-H_6_L-Cu_2_ xerogel obtained from EtOH/H_2_O (1/1, *v*/*v*).

The quantitative gelation investigation of (*R*)-H_6_L with Cu^2+^ was performed by gradually adding Cu^2+^ (0–4 equiv.) to (*R*)-H_6_L (4.5 mmol in EtOH/H_2_O 1/1, *v*/*v*) via the inverted vial method (Figure S8). A suspension was formed with 0.05 equiv. of Cu^2+^, and an unstable gel with weak fluidity was produced with 0.1 equiv. of Cu^2+^. Starting from 0.2 equiv. of Cu^2+^ addition, the gel became stable with no fluidity. The quantitative investigation of (*R*)-H_6_L with Cu^2+^ in a gel was performed by gradually adding Cu^2+^ to an EtOH/H_2_O solution of (*R*)-H_6_L using CD spectroscopy (Figure 3a). (*R*)-H_6_L showed negative CD signals in the region of 270–380 nm, which were caused by the chiral binaphthalene group and benzene group. With the continuous addition of Cu^2+^, this negative CD band was first significantly reduced with 0.05 equiv. of Cu^2+^ and then reversed to positive CD signals at 290 nm and 354 nm with 0.1 equiv. of Cu^2+^, which meant that Cu^2+^ changed from its previous self-assembly structure with a negative CD band and formed a new chiral assembly structure (*R*)-H_6_L-Cu^2+^ with a positive CD band. When the Cu^2+^ addition was in the range of 0.2 equiv. to 4 equiv., the CD spectra exhibited similar patterns, and the CD intensity at 340 nm and 365 nm gradually increased from 0.2 equiv. to 2 equiv. and then decreased steadily, which indicated a stable gel and implies that the coordinated stoichiometry for Cu^2+^ to (*R*)-H_6_L is 2:1. The gel with 2 equiv. Cu^2+^ was named (*R*)-H_6_L-Cu_2_ and that with 0.2 equiv. Cu^2+^ was named (*R*)-H_6_L-Cu_0.2_.

^1^H NMR studies of (*R*)-H_6_L with different equivalents of Cu^2+^ (0–3 equiv.) in EtOD-*d_6_* were also conducted (Figure 3b), which showed that the signals of all aromatic protons had an upfield shift with the addition of Cu^2+^ from 0.2 to 2 equiv. and remained unchanged from 2 to 3 equiv., confirming that the coordination ratio for (*R*)-H_6_L: Cu^2+^ is 1:2. The observed upfield shifts in the chemical shifts of all aromatic protons could be attributed to π–π stacking interactions between aromatic rings, which were likely induced by metal-coordination-driven supramolecular assembly. The coordination of Cu^2^⁺ with (*R*)-H_6_L promoted structural organization, facilitating the closer and more ordered alignment of the aromatic moieties. This π–π stacking enhanced the ring current shielding effect, thereby shifting the aromatic proton resonances to higher fields in the ^1^H NMR spectrum.

The FT-IR spectrum of the xerogel of (*R*)-H_6_L-Cu_2_ was compared with that of the powder of (*R*)-H_6_L (Figure 3c). The FT-IR analysis revealed a significant decrease in intensity for the carboxylic acid C=O stretching vibration peak at 1699 cm⁻^1^ and the C-O stretching vibration peak at 1230 cm⁻^1^, while the O-H stretching vibration peak in phenolic hydroxyl groups at 3430 cm^−1^ remained unchanged, indicating that carboxylic acid groups coordinate with Cu^2+^ while phenolic hydroxyl groups do not. The absence of hydroxyl group coordination can likely be attributed to the steric hindrance exerted by substituents located at the ortho positions of the binaphthyl framework, which restricts access to the metal center.

Transmission electron microscopy (TEM) and scanning electron microscopy (SEM) were utilized to characterize the morphology of the gels. TEM showed that both the (*R*)-H_6_L-Cu_0.2_ gel and (*R*)-H_6_L-Cu_2_ gel assembled into a cross-linked fibrous structure; the former had small spherical aggregates attached to the nanofibers, while the latter had a more homogeneous and dense fiber network structure (Figure 3d,e). The TEM data of dilute (*R*)-H_6_L (0.45 mM) and (*R*)-H_6_L (0.45 mM) with 2 equiv. Cu^2+^ in EtOH/H_2_O (1/1, *v*/*v*) were also recorded for comparison. In a dilute solution, (*R*)-H_6_L presented as nanovesicles or spheres, and the addition of 2 equiv. Cu^2+^ strung these particles together (Figure S9). The SEM of the (*R*)-H_6_L-Cu_0.2_ xerogel revealed that there was a large bundle of fine fibers arranged together, with some small spheres adhered to them, while it formed relatively coarse and straight fibers in the (*R*)-H_6_L-Cu_2_ xerogel. We can conclude that, under the gelation conditions, (*R*)-H_6_L coordinated with Cu^2+^ to form a cross-linked fiber network structure, resulting in the formation of a gel. (*R*)-H_6_L with 0.2 equiv. of Cu^2+^ partially assembled into fibers due to the incomplete coordination, and the remaining molecules aggregated into spheres to connect with the fibers, while 2 equiv. of Cu^2+^ rendered the fibrosis more complete.

**Figure 4:**
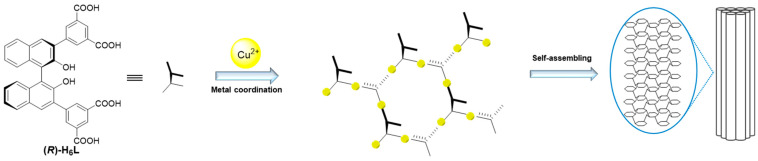
Schematic illustration of the gelation of (R)-H_6_L.

According to the CD, NMR, and FT-IR experiments, we confirm the coordination between Cu^2+^ and the carboxyl groups in compound (*R*)-H_6_L, establishing a metal-to-ligand stoichiometric ratio of 2:1 (M:L = 2:1). In this case, it forms a three-dimensional network structure of (*R*)-H_6_L-Cu^2+^, which is supported by SEM and TEM. In other words, Cu^2+^ connects the (*R*)-H_6_L molecules in series to form long chains; meanwhile, the spare carboxyl groups of ligands on the different chains are connected through Cu^2+^ [22]. Then, self-assembly dominated by hydrogen bond interactions and π–π stacking results in the formation of gels (Figure 4). This model explains the opposite chirality and weak intensity in the CD spectrum. The antiparallel arrangement of (*R*)-H_6_L modified the inherent chirality of the self-assembly originally formed by hydrogen-bonding interactions and π–π stacking interactions, while the network structure led to the diminished chiral amplification of the assembly. This model further explains the gelation mechanism with low equiv. of metal ions. Not all carboxyl groups of (*R*)-H_6_L participate in metal coordination, and a minimal amount of Cu^2^⁺ (0.2 equiv.) suffices to induce structural assembly, while higher concentrations (2 equiv.) promote more complete planar-directed assembly. At the molecular level, this enhanced coordination manifests as increased structural stability, as evidenced by the CD and NMR titration experiments. Macroscopically, it results in thicker fibrous networks, as directly visualized through SEM.

### 2.3. Chemoselective and Enantioselective Recognition of Tartaric Acid by Gel Collapse

**Figure 5:**
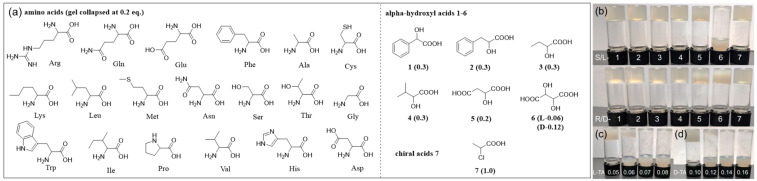
(**a**) Different amino acids, α-hydroxy acids, and no-hydroxy acids with their lowest eq. for gel collapse. (**b**) Response behavior of the (*R*)-H_6_L-Cu_0.2_ gel (4.5 mM) in EtOH/H_2_O (1/1, *v*/*v*) toward 0.1 eq of chiral hydroxy acids. (*R*)-H_6_L-Cu_0.2_ gel in the presence of (**c**) L-TA (0.05 eq., 0.06 eq., 0.07 eq., 0.08 eq.) and (**d**) D-TA (0.10 eq., 0.12 eq., 0.14 eq., 0.16 eq.) with different equivalents.

(*R*)-H_6_L obtained stable chiral MOGs with Cu^2+^ through the coordination of carboxyl groups. To explore its potential for enantiomer recognition, we systematically investigated its response to the enantiomers of various chiral acids, including 19 common amino acids, α-hydroxyl carboxylic acids **1**–**6**, and regular chiral acid 2-chloropropanoic acid **7** (Figure 5a). In order to achieve more sensitive recognition, the equivalent of Cu^2+^ was reduced to the minimum of 0.2, which still formed a stable gel with (*R*)-H_6_L (4.5 mM in EtOH/H_2_O 1/1, *v*/*v*) (Figure 5a and Figure S8). Chiral acids of different equivalents (0.05–0.5 equiv.) were added to the (*R*)-H_6_L-Cu_0.2_ gel, and it was found that all tested carboxylic acids induced gel collapse, except 2-chloropropanoic acid **7**, proving that amino groups or hydroxyl groups at the α-position play an important role in gel collapse (Table S2). The abilities of various chiral acids to induce gel collapse are also different. All 19 amino acids collapsed the gel at 0.2 equivalents, while mono hydroxyl acids **1**–**4** caused gel collapse at 0.3 equivalents, ascribed to the stronger coordinating ability of amino groups with Cu^2+^ than hydroxyl groups. Malic acid **5** features an extra carboxylic acid group; it disrupted the gel at lower equivalents (0.2 equiv.) than mono acids **1**–**4**. The gel responses caused by amino acids and hydroxyl acids **1**–**5** are not enantioselective. Of all tested chiral acids, tartaric acid (TA) **6**, featuring two hydroxyl groups and two carboxylic groups, exhibited the most unique and sensitive gel response, exhibiting enantioselective gel collapse at 0.1 equiv. with the L-enantiomer only (Figure 5b).

Therefore, the response behavior of the (*R*)-H_6_L-Cu_0.2_ gel (4.5 mM) in EtOH/H_2_O (1/1, *v*/*v*) toward 0.1 equiv. of 26 chiral carboxylic acids was studied, and it was found that only L-TA caused gel collapse, while D-TA and both enantiomers of the remaining 25 chiral acids caused no change to the gel, indicating that (*R*)-H_6_L-Cu_0.2_ has both chemoselective and enantioselective recognition abilities toward L-TA by simple visual gel collapse. The disrupting capacity of TA on the (*R*)-H_6_L-Cu_0.2_ gel was further carefully examined through a series of concentration gradients. The gel underwent rapid collapse upon the addition of merely 0.06 equivalents of L-TA (Figure 5c), in stark contrast to its D-enantiomer, which required twice the amount to induce similar gel collapse (Figure 5d). The enantiomer (*S*)-H_6_L was synthesized and exhibited identical gelation properties. The resulting (*S*)-H_6_L-Cu_0.2_ gel demonstrated enantioselective recognition toward D-TA, undergoing collapse at a remarkably low concentration of 0.06 equiv. In contrast, the L-enantiomer required twice the amount (0.12 equiv.) to induce gel collapse, mirroring the chiral recognition behavior observed in its (*R*)-H_6_L enantiomer.

**Figure 6:**
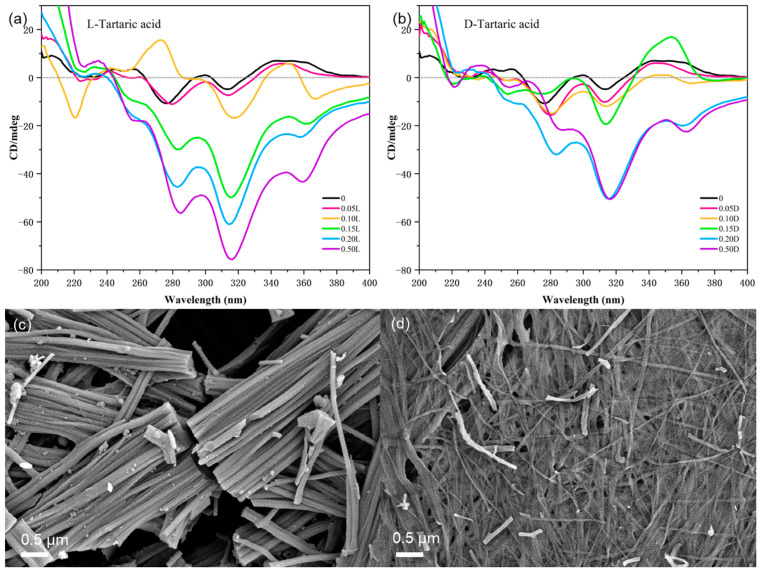
CD spectra of (*R*)-H_6_L-Cu_0.2_ gel (4.5 mM) + (**a**) L-TA and (**b**) D-TA with different equivalents in EtOH/H_2_O (1/1, *v*/*v*). SEM image of (*R*)-H_6_L-Cu_0.2_ (4.5 mM) after adding 0.1 eq. (**c**) L-TA and (**d**) D-TA obtained from EtOH/H_2_O (1/1, *v*/*v*).

In order to study the process of (*R*)-H_6_L-Cu_0.2_’s enantioselective recognition of tartaric acid, the CD spectra were measured (Figure 6). The addition of 0.05 equiv. of L-TA had no effect on the gel and did not change the CD signal. The gel collapsed after adding 0.1 equiv. of L-TA, with a new negative peak appearing at 368 nm and a positive peak appearing at 270 nm (Figure 6a), indicating that L-TA entered the chiral sites of (*R*)-H_6_L-Cu^2+^ and formed an intermediate structure. With the addition of more than 0.15 equiv. of L-TA, the CD spectra recovered to the CD signals of (*R*)-H_6_L only, showing that L-TA completely displaced Cu^2+^ from (*R*)-H_6_L via metal coordination. For D-TA, the addition of 0.05 equiv. did not change the CD signal in the same way. The CD signal of (*R*)-H_6_L-Cu _0.2_ with 0.15 equiv. D-TA was similar to that of (*R*)-H_6_L with 0.1 equiv. Cu^2+^ (Figure 3a). Excessive D-TA restored the negative CD signal and maintained it (Figure 6b), which could indicate that D-TA competed with (*R*)-H_6_L for the coordination of Cu^2+^.

Malic acid (MA) **5**, with fewer hydroxyl groups, was also measured (Figure S11). It was found that both L-MA and D-MA had the same effect on the (*R*)-H_6_L-Cu_0.2_ gel, indicating that the additional hydroxyl group played an important role in chiral recognition. Meanwhile, succinic acid, with no hydroxyl groups, and 2,3-butanediol, with no carboxyl groups, were also tested (Figure S12). Neither of them had an effect on the CD signal or caused the gel to collapse with 0.2 equiv. addition, proving that visual recognition via the collapse of the gel requires the interaction of hydroxyl and carboxyl groups.

Scanning electron microscopy (SEM) studies provided the direct visualization of the distinct morphological impacts exerted by D- and L-TA enantiomers on the (*R*)-H_6_L-Cu_0.2_ gel. As shown in Figure 6c, the addition of 0.1 equiv. of L-TA cracked the fibers and thereby collapsed the gel. In contrast, D-TA formed some broken fibers while retaining the cross-linked fibrous structure (Figure 6d), which was similar to the effects seen in (*R*)-H_6_L-Cu_0.1_ (Figure S13). This similarity suggests that D-TA destabilizes the gel by competitively coordinating with Cu^2^⁺, thereby disrupting the (*R*)-H_6_L-Cu^2+^ gel architecture.

The formation constants were determined to compare the coordination abilities of tartaric acid and (*R*)-H_6_L with Cu^2+^. By monitoring the chemical shift changes of specific protons in (*R*)-H_6_L as a function of the Cu^2^⁺ concentration, the fitting yielded a high correlation coefficient (R^2^ = 0.99), and the calculated formation constant (K) was determined to be 2.45 × 10^3^ M^−2^ with lgK = 3.39 (Figures S14 and S15). According to reference [23], under neutral conditions, tartaric acid coordinates with Cu^2+^ in a 1:2 ratio, and the corresponding lgK as a critical stability constant is 5.11. These data demonstrate that tartaric acid coordinates more readily with Cu^2+^ compared to (*R*)-H_6_L.

Therefore, a possible mechanism for the chemoselective and enantioselective gel collapse was proposed. Carboxylic acid with alpha-hydroxyl or amino groups is more likely to coordinate with Cu^2+^ by chelation than carboxyl groups alone. It can compete with (*R*)-H_6_L for Cu^2+^ coordination and then cause the gel to collapse. It is easier for chirality-matched L-TA to enter the chiral pores or chiral sites of the (*R*)-H_6_L-Cu^2+^ gel and capture Cu^2+^ [21], while D-TA with chirality mismatch requires a greater amount to compete with (*R*)-H_6_L for Cu^2+^ coordination, thus realizing enantioselective gel collapse.


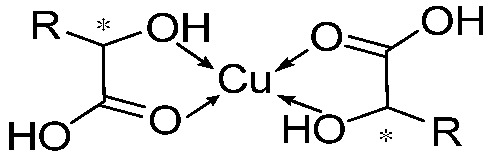


## 3. Conclusions

In summary, a chiral supramolecular gelator, (*R*)-H_6_L, was successfully synthesized, exhibiting a lower critical gelation concentration compared to (*R*)-H_3_L. In an EtOH/H_2_O (1:1, *v*/*v*) system, (*R*)-H_6_L selectively formed a gel with Cu^2+^ at a low threshold of 0.2 equivalents, facilitated by its unique coordination–assembly structure. The resulting metal–organic gel, (*R*)-H_6_L-Cu_0.2_, exhibited highly sensitive chemoselective and enantioselective collapse in response to tartaric acid, achieving a low detection limit of 0.06 equivalents. This work presents a device-free, visual recognition strategy for both metal ions (Cu^2+^) and TA enantiomers, offering valuable insights for visual sensing applications and serving as a promising model for future chiral sensor design.

## 4. Materials and Methods

### 4.1. Materials

(*R*)-2,2′-(2,2′-bis(methoxymethoxy)-[1,1′-binaphthalene]-3,3′-diyl)bis(4,4,5,5-tetramethyl-1,3,2-dioxaborolane) was synthesized as previously described [19]. All chemicals and reagents were of analytical grade and used without further purification. All solvents for the optical spectroscopic studies were of high-performance liquid chromatography grade. Analytical-grade solvents and all reagents were purchased from Energy Chemical Co., Ltd. (Shanghai, China) and Sigma-Aldrich (Shanghai, China).

### 4.2. Instruments

A Bruker AM400 NMR spectrometer was used for ^1^H NMR and ^13^C NMR analysis. High-resolution mass spectra (HRMS) were obtained by using a Bruker Daltonics Bio TOF mass spectrometer. Circular dichroism (CD) absorption spectra were measured with the Applied Photophysics Chirascan (Applied Photophysics, Leatherhead, UK). The FT-IR (KBr pellet) spectra were obtained (400–4000 cm^−1^ region) by a Thermo Fisher Scientific Nicolet iS20 (Thermo Fisher Scientific, Waltham, MA, USA) spectrometer. Scanning electron microscopy (SEM) images were obtained on a ZEISS Sigma 360 (Carl Zeiss, Oberkochen, Germany). Transmission electron microscopy (TEM) images were recorded on a Talos F200S instrument (Thermo Fisher Scientific, Waltham, MA, USA).

### 4.3. Gelation Test by the Inverted Vial Method

(*R*)-H_6_L was dissolved in various solvents by heating within sample vials. The samples were then cooled to room temperature, and gel formation was confirmed via the inverted vial method. The gel was considered formed if the (*R*)-H_6_L and solvent mixture remained immobilized at the vial bottom, with no flow observed. For vials exhibiting gelation, additional solvent was incrementally added until a stable gel could no longer be obtained. The concentration at this critical point was defined as the CGC of (*R*)-H_6_L in the tested solvent system.

### 4.4. Visual Metal Sensing of (R)-H_6_L

To a solution of (*R*)-H_6_L (14.8 mM) in EtOH (0.30 mL), a solution of metal salt (2 equiv.) in water (0.30 mL) was added. The mixture was heated to ensure complete dissolution and homogenization. After cooling to room temperature, gel stability was assessed using the inverted vial method. Control experiments were conducted with ethanol as the solvent.

### 4.5. Influence of pH on Gelation of (R)-H_6_L-Cu^2+^ Gel

To a solution of (*R*)-H_6_L (18 mM) in EtOH (0.15 mL), a solution of metal salt (2 equiv.) in EtOH (0.15 mL) was added. A series of aqueous solutions with varying pH gradients were formulated using sodium hydroxide (NaOH) and hydrochloric acid (HCl). The equal-volume mixture was heated to ensure complete dissolution and homogenization. After cooling to room temperature, gel stability was assessed using the inverted vial method. Control experiments were conducted with ethanol as the solvent.

### 4.6. Visual Chiral Sensing of (R)-H_6_L-Cu^2+^ Gel

To a solution of (*R*)-H_6_L (4.5 mM) in EtOH (0.20 mL), a solution of hydroxy acid (0.1 equiv.) in EtOH (0.10 mL) and a solution of copper(II) chloride (0.2 equiv.) in water (0.30 mL) were added. The mixture was heated to ensure complete dissolution and homogenization. The system was maintained at room temperature for 4 h, and gel stability was assessed using the inverted vial method. Control experiments were conducted with ethanol as the solvent.

### 4.7. Microstructure

The prepared gel was converted into a xerogel via freeze-drying, pasted onto a conductive adhesive, and placed on a stage, and the surface was sprayed with gold to observe the morphology by SEM. After aging for 24 h, the prepared gel was diluted to 0.45 mM with the corresponding solvent system; then, the sample was dropped onto a carbon-coated copper mesh (300 mesh), and the morphology was observed after vacuum-drying by TEM.

### 4.8. CD Spectrum

Samples diluted to different concentrations and gel samples were prepared and tested on the spot. The preparation method for gel samples was as follows: 300 µL (*R*)-H_6_L EtOH solution was placed into a 2 mL sample vial and then 300 µL metal aqueous solution was added. The sample preparation method was consistent for all concentrations. The optical path length of the quartz cuvette used for all samples was 0.1 mm.

## Figures and Tables

**Table 1:** Gelation properties of (*R*)-H_6_L ^a^.

Solvent	Result	Solvent	Result	Solvent	Result
CHCl_3_	I	CH_3_COOCH_2_CH_3_	S	Acetone/H_2_O	S
CH_2_Cl_2_	I	THF	S	THF/H_2_O	S
n-Hexane	I	EtOH	S	1,4-Dioxane/H_2_O	S
CH_3_CN	I	MeOH	S	*i*-PrOH/H_2_O	S
(CH_3_CH_2_)_2_O	I	DMF	S	EtOH/H_2_O	PG
Toluene	I	1,4-Dioxane	S	MeOH/H_2_O	PG
H_2_O	I	DMSO	S	DMF/H_2_O (1/1, *v*/*v*)	G (20.3 mM)
Acetone	S	*i*-PrOH	S	DMSO/H_2_O (1/1, *v*/*v*)	G (14.8 mM)

^a^ I: insoluble, G: stable gel, PG: partial gel, S: sol. In parentheses, the critical gelation concentration (CGC) and the volume ratio of organic solvent and water required to form stable water-induced gels, respectively, are given.

## Data Availability

The original contributions presented in this study are included in the article/Supplementary Materials. Further inquiries can be directed to the corresponding author.

## Supplementary Materials

The following supporting information can be downloaded at: https://www.mdpi.com/article/10.3390/gels11050340/s1, Figures S1–S5: ^1^H NMR spectra, ^13^C NMR spectra and mass spectrum; Figure S6: The effect of adding water on the gelation of (*R*)-H_6_L in DMSO/H_2_O; Figure S7: The effect of adding 2 equiv. CuCl_2_ on the gelation of 14.8 mM (*R*)-H_6_L in different solvents mixed with water (1/1, *v*/*v*); Figure S8: The effect of adding CuCl_2_ to 4.5 mM (*R*)-H_6_L in EtOH/H_2_O; Figure S9: TEM; Figure S10: (*S*)-H_6_L-Cu_0.2_ gel in the presence of TA with different equivalents; Figure S11: CD spectra of (*R*)-H_6_L-Cu_0.2_ gel (4.5 mM) with the addition of malic acid in EtOH/H_2_O (1/1, *v*/*v*); Figure S12: CD spectra of (*R*)-H_6_L-Cu_0.2_ gel (4.5 mM) with the addition of succinic acid and 2,3-butanediol in EtOH/H_2_O (1/1, *v*/*v*); Figure S13: SEM image of (*R*)-H_6_L-Cu_0.1_; Figure S14: ^1^H NMR spectra (DMSO-*d*_6_) of (*R*)-H_6_L with different equivalents of Cu^2+^; Figure S15: Determination of host-guest binding constants by analyzing proton H^1^ shifts in ^1^H NMR titration; Table S1. Gelation properties of (*R*)-H_6_L; Table S2: Gelation properties of (*R*)-H_6_L with different equiv. of acids.
