# Supplementary material for: Tandem Visual Recognition of Cu2+ and Chiral Tartaric Acid by Sequence Gel Formation and Collapse
Source: Gels. 2025 May 1;11(5):340. doi: 10.3390/gels11050340 (PMC12111522; doi:10.3390/gels11050340)
Supplement: Supplementary file 1 [file gels-11-00340-s001.zip › gels-3594355-supplementary.pdf]

Electronic Supplementary Information

Tandem visual recognition of Cu^{2+} and chiral tartaric acid by sequence gel formation and collapse

Jian Zeng, Yixuan Jiang, Xiao-Qi Yu, and Shanshan Yu *

Key Laboratory of Green Chemistry and Technology, Ministry of Education, College of Chemistry, Sichuan University, Chengdu 610064, China.

*Corresponding author e-mail: yushanshan@scu.edu.cn (S.Yu)

Table of Contents

1. Synthesis of (*R*)-H_6L

2. Supplementary Figures and Tables

1. Synthesis of (S)-H_6L

Synthesis of **2**

A 500 mL flame-dried round-bottom flask was charged with **1** (2.0 g, 3.2 mmol), dimethyl 5-bromoisophthalate (2.18 g, 8 mmol) and tetrakis triphenylphosphine palladium (0.37 g, 0.32 mmol), degassed and refilled with argon for three times. Then degassed THF (200 mL) and 2 M K_2CO_3 solution (30 mL, aq.) was added, and the suspension was heated under reflux conditions for 24 hours. After cooling to room temperature, the resulting mixture was filtered with diatomite, extracted with EtOAc (100 mL ×3), dried over Na_2SO_4, and concentrated under reduced pressure. The crude product was purified by silica gel column chromatography eluting with hexane/ EtOAc to afford **2** (2.1 g) as a light yellow solid in 86% yield. ^1H NMR (400 MHz, Chloroform-*d*) δ 8.72 (s, 2H), 8.61 (s, 4H), 8.01 (s, 2H), 7.93 (d, J = 8.1 Hz, 2H), 7.47 (t, J = 6.7 Hz, 2H), 7.35 (m, 2H), 7.30 (d, J = 8.4 Hz, 2H), 4.45 – 4.37 (m, 4H), 3.99 (s, 12H), 2.44 (s, 6H). ^{13}C NMR (101 MHz, Chloroform-*d*) δ 166.4, 151.5, 140.1, 135.1, 134.1, 133.8, 131.1, 130.9, 130.9, 129.7, 128.2, 127.1, 126.5, 125.7, 99.0, 56.3, 52.6. HR-MS (ESI+) calculated for $C_{44}H_{38}O_{12}Na^+$ (M+Na^+) 781.2255, found 781.2252.

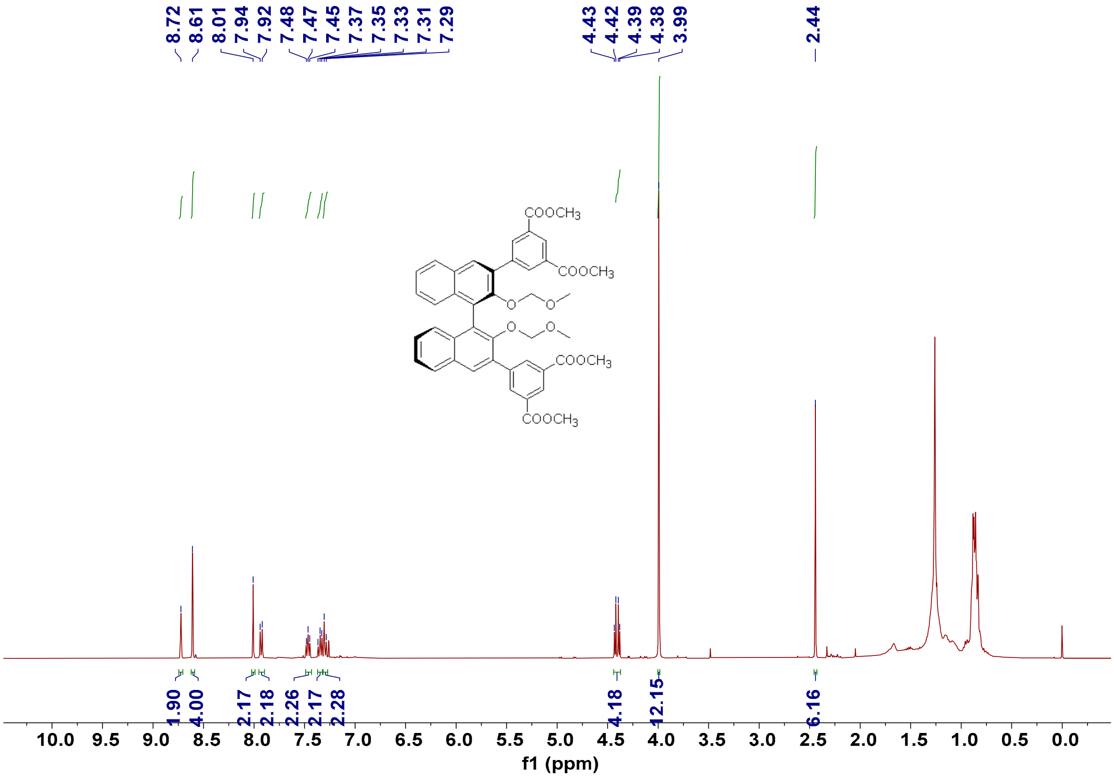

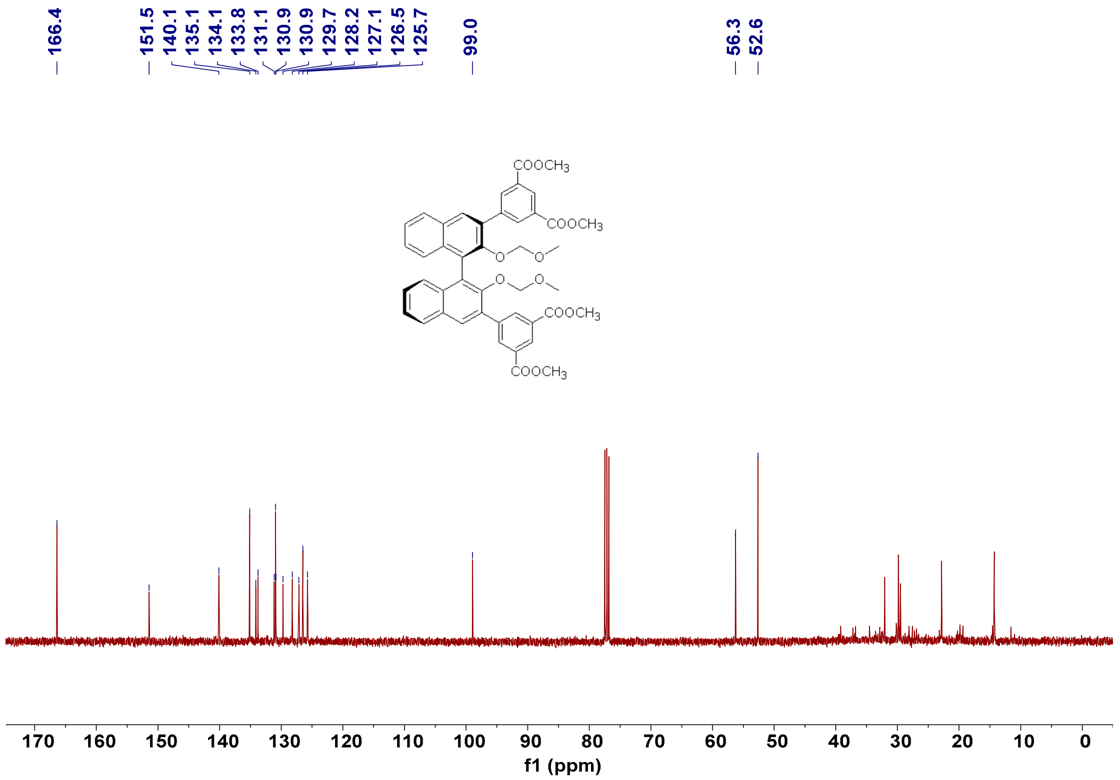

Figure S1. ^{1}H NMR (400 MHz, Chloroform-*d*) and ^{13}C NMR (101 MHz, Chloroform-*d*) spectra of **2**.

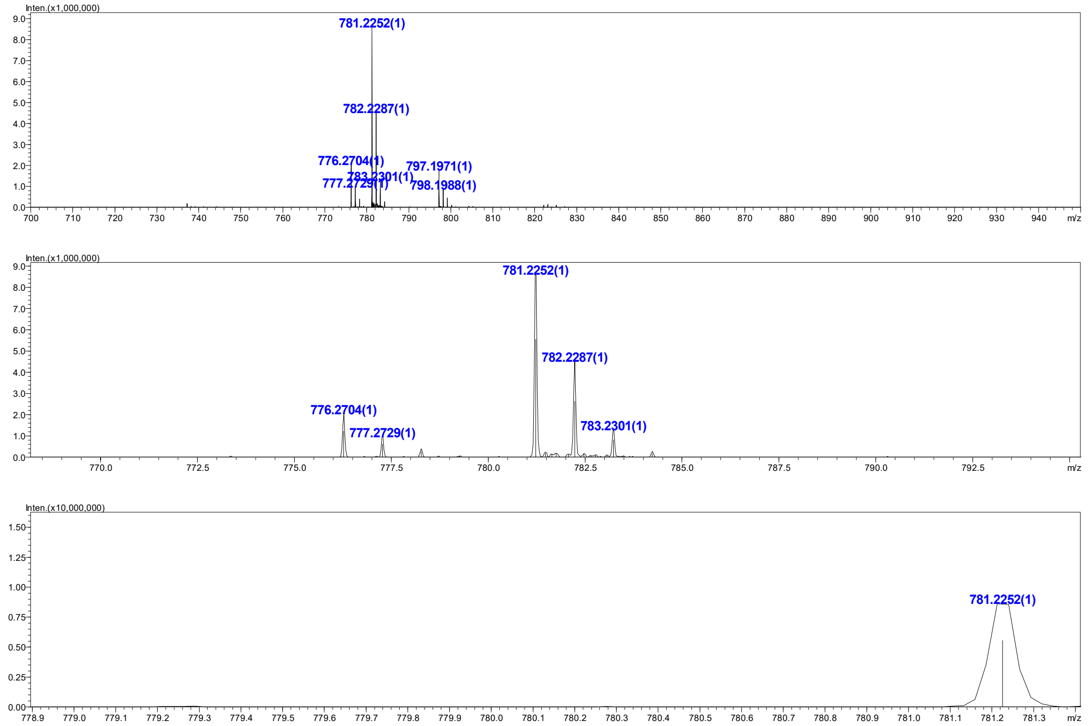

Figure S2. HRMS spectra of **2** (ESI^{+}).

Synthesis of **3**

To a solution of **2** (2.1 g, 2.77 mmol) in CH_2Cl_2 (200 mL) was added trifluoroacetic acid (20 mL). After stirring at room temperature for 8 h, the reaction mixture was quenched by saturated aqueous sodium bicarbonate solution, extracted with CH_2Cl_2 (100 mL ×3), dried over Na_2SO_4, and

concentrated under reduced pressure. The crude product was dissolved in a small amount of CH_2Cl_2 and a large amount of hexane, stirred at room temperature overnight and filtered to give **3** (1.8 g) as a light orange solid in 97% yield. ^{1}H NMR (400 MHz, Chloroform-*d*) δ 8.69 (s, 2H), 8.28 (s, 4H), 7.83 (d, *J* = 8.1 Hz, 2H), 7.61 (s, 2H), 7.36 (t, *J* = 7.4 Hz, 2H), 7.28 (d, *J* = 7.1 Hz, 2H), 7.10 (d, *J* = 8.4 Hz, 2H), 6.28 (s, 2H), 3.81 (s, 12H). ^{13}C NMR (101 MHz, Chloroform-*d*) δ 166.3, 150.3, 138.7, 134.9, 133.9, 131.6, 130.4, 129.5, 129.3, 128.7, 128.6, 127.8, 124.5, 124.4, 112.6, 52.5.

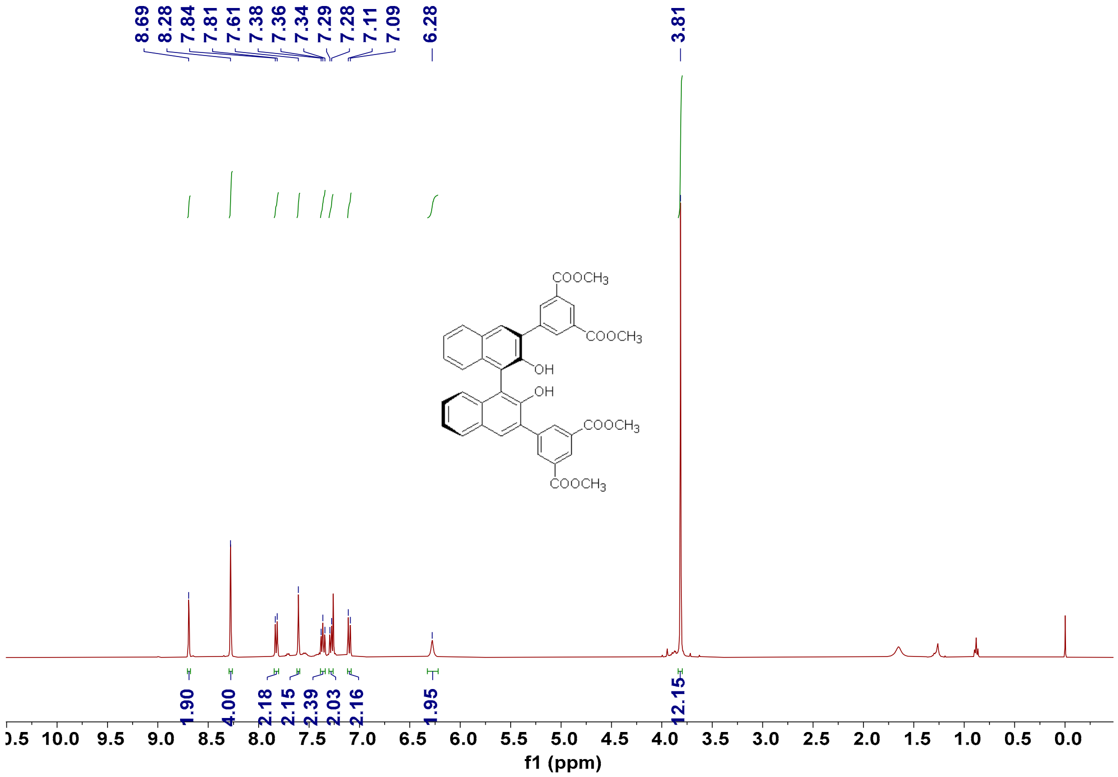

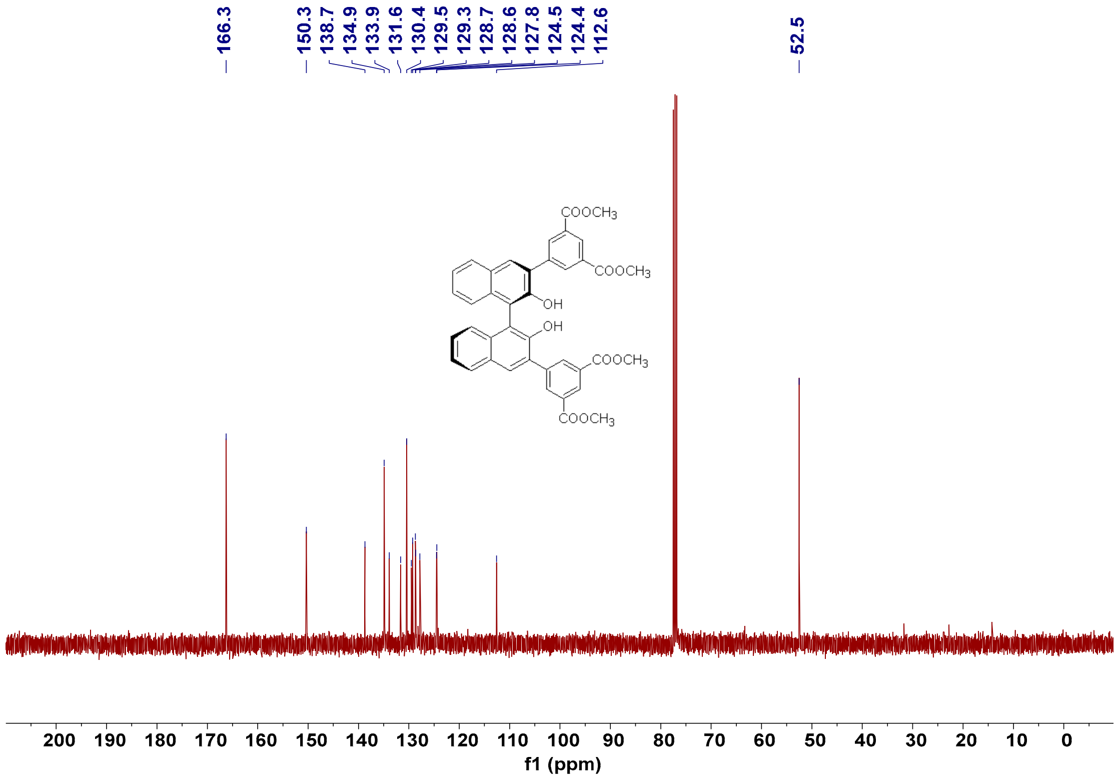

Figure S3. ^{1}H NMR (400 MHz, Chloroform-*d*) and ^{13}C NMR (101 MHz, Chloroform-*d*) spectra of **2**

Synthesis of **(*R*)-H_6L**

To a solution of **3** (1.8 g, 2.68 mmol) in THF (125 mL) was added 3 M KOH solution (125 mL). After stirring at 40 °C for 24 h, the solution was cooled to room temperature and acidified to pH ~ 1 with 2 M HCl, extracted with EtOAc (100 mL ×3), dried over Na_2SO_4, and concentrated under reduced pressure to give **(*R*)-H_6L** (1.57 g) as a white solid in 90% yield. ^{1}H NMR (400 MHz, DMSO-d_6) δ 8.63 (s, 2H), 8.54 (s, 2H), 8.53 (s, 2H), 8.51 (s, 2H), 8.09 (s, 2H), 8.02 (d, J = 8.0 Hz, 2H), 7.33 (t, J = 7.2 Hz, 2H), 7.26 (t, J = 8.0 Hz, 2H), 6.95 (d, J = 8.0 Hz, 4H). ^{13}C NMR (101 MHz, DMSO-d_6) δ 167.24, 151.65, 140.13, 135.06, 134.80, 134.36, 131.60, 130.71, 130.33, 129.07, 128.86, 127.29, 126.95, 124.53, 124.32, 123.82, 123.45, 115.23. HR-MS (ESI^+) calculated for $C_{36}H_{22}O_{10}Na^+$ ($M+Na^+$) 637.1105, found 637.1107.

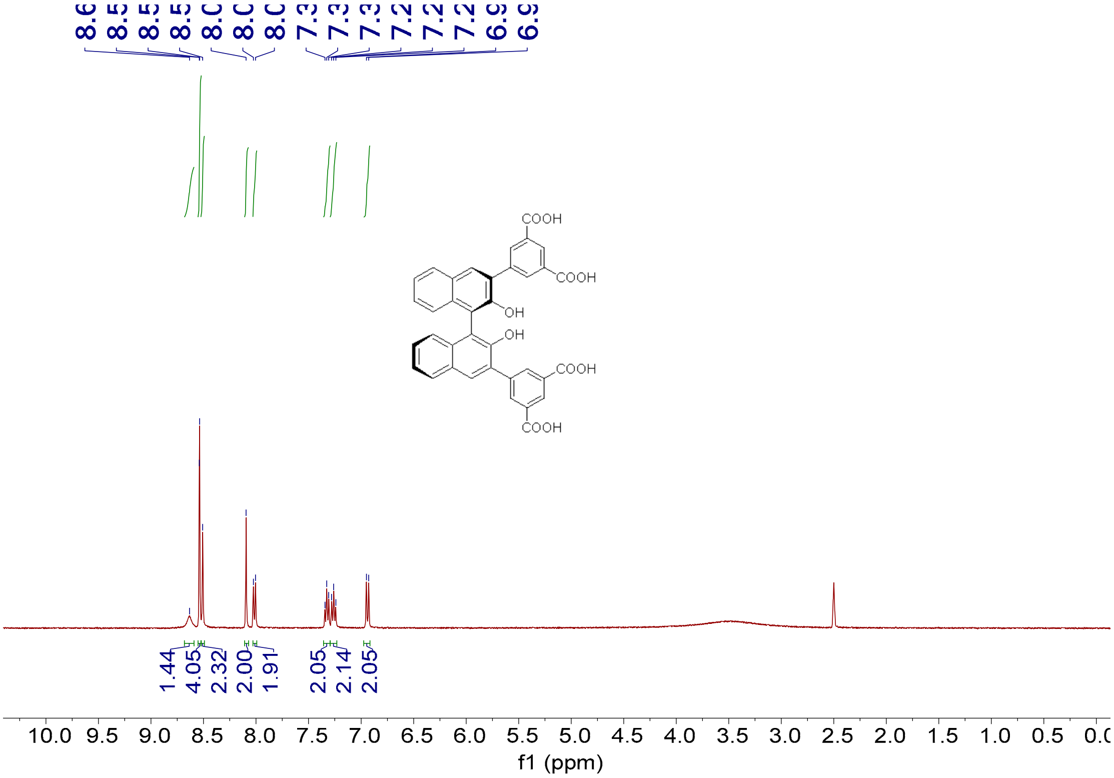

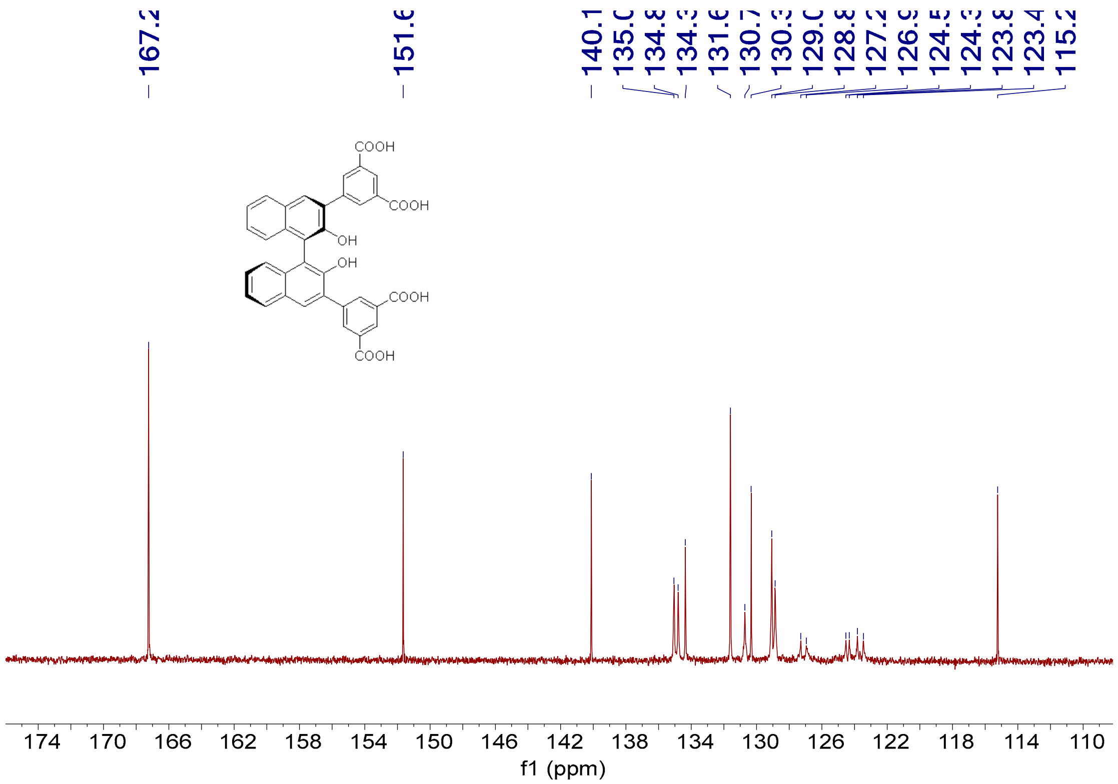

Figure S4. ^{1}H NMR (400 MHz, DMSO-d_6) and ^{13}C NMR (101 MHz, DMSO- d_6) spectra of **(*R*)-H$_6$L**.

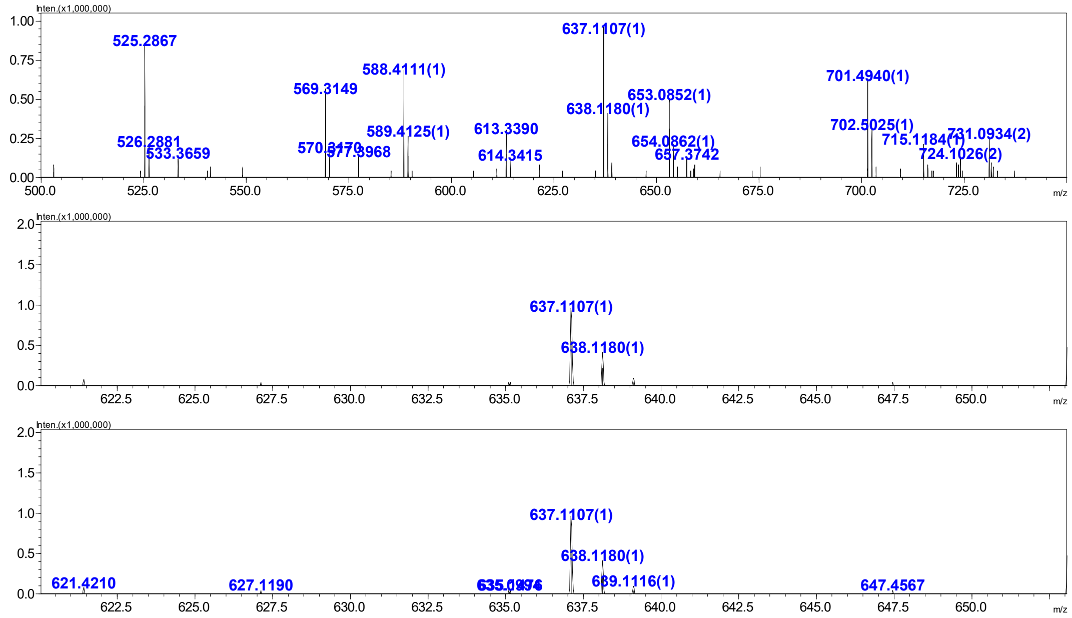

Figure S5. HR-MS spectra of **(*R*)-H$_6$L** (ESI^{+}).

2. Supplementary Figures and Tables

Table S1. Gelation properties of (*R*)-H$_6$L^{a}

Solvent	Result	Solvent	Result	Solvent	Result
$CHCl_3$	I	$CH_3COOCH_2CH_3$	S	Acetone/H_2O	S
CH_2Cl_2	I	THF	S	THF/H_2O	S
n-Hexane	I	EtOH	S	1,4-Dioxane/H_2O	S
CH_3CN	I	MeOH	S	*i*-PrOH/H_2O	S

$(CH_3CH_2)_2O$	I	DMF	S	EtOH/H_2O	PG
Toluene	I	1,4-Dioxane	S	MeOH/H_2O	PG
H_2O	I	DMSO	S	DMF/H_2O (1/1, v/v)	G (20.3 mM)
Acetone	S	*i*-PrOH	S	DMSO/H_2O (1/1, v/v)	G (14.8 mM)

[a]I: insoluble, G: stable gel, PG: partial gel, S: sol. In parentheses, critical gelation concentration (CGC) and the volume ratio of organic solvent and water required to form stable water-induced gels, respectively, are given.

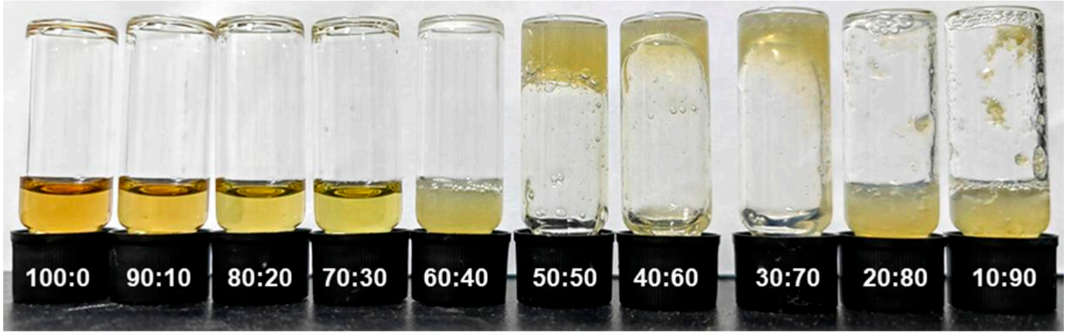

Figure S6. The effect of adding water on the gelation of (*R*)-H_6L in DMSO/H_2O. From left to right: the ratio of water (f_w) was increased from 0 to 100%.

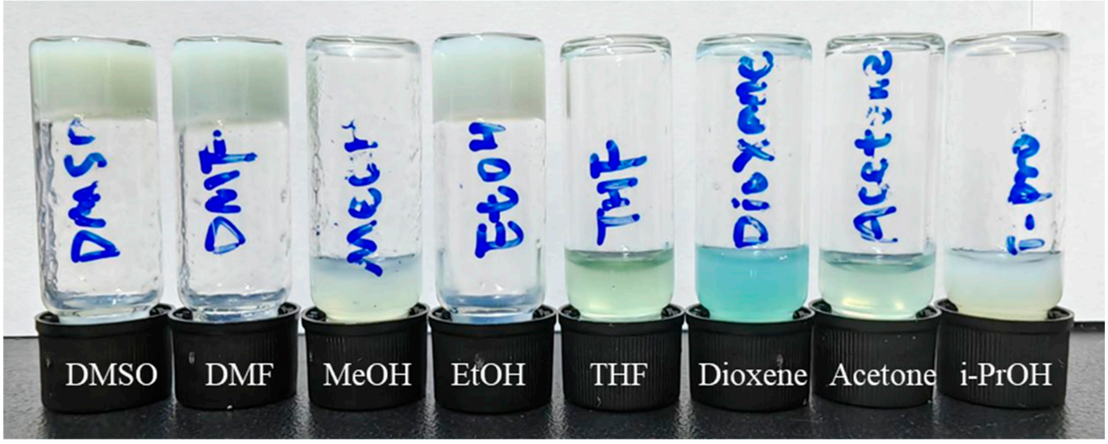

Figure S7. The effect of adding 2 equiv. $CuCl_2$ on the gelation of 14.8 mM (*R*)-H_6L in different solvents mixed with water (1/1, v/v)

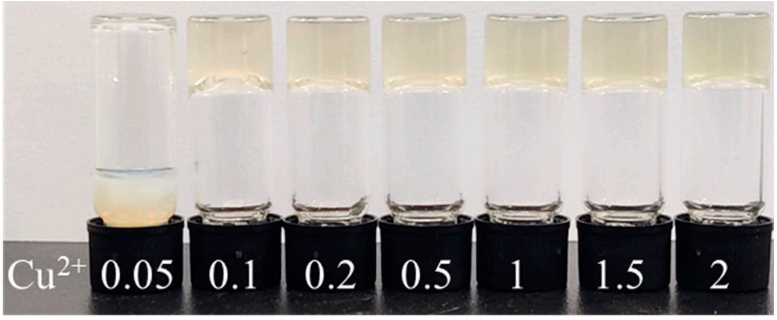

Figure S8. The effect of adding $CuCl_2$ to 4.5 mM (*R*)-H_6L in EtOH/H_2O. The gel of 0.1 equiv. Cu^{2+} wasn't stable.

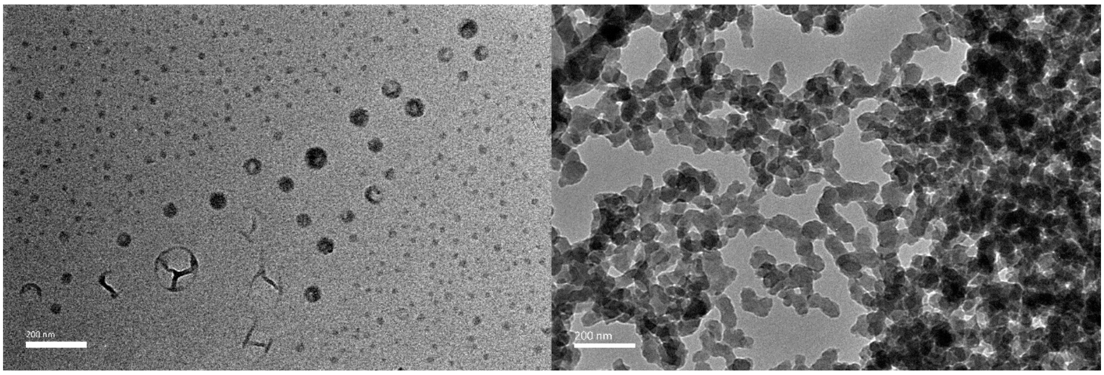

Figure S9. TEM image of (c) (*R*)-H_6L(0.45 mM) and (d) (*R*)-H_6L with 2 equiv. Cu^{2+} in EtOH/H_2O (1/1, v/v).

Table S2. Gelation properties of (*R*)-H_6L with different equiv. of acids[b]

	0.05	0.1	0.15	0.2	0.3	0.5	1.0	2.0
Phe	√	√	√	×	×	×	×	×
Asp	√	√	√	×	×	×	×	×
Glu	√	√	√	×	×	×	×	×
Ala	√	√	√	×	×	×	×	×
Thr	√	√	√	×	×	×	×	×
Ser	√	√	√	×	×	×	×	×
Pro	√	√	√	×	×	×	×	×
Val	√	√	√	×	×	×	×	×
His	√	√	√	×	×	×	×	×
Asn	√	√	√	×	×	×	×	×
Gln	√	√	√	×	×	×	×	×
Leu	√	√	√	×	×	×	×	×
Ile	√	√	√	×	×	×	×	×
Arg	√	√	√	×	×	×	×	×
Trp	√	√	√	×	×	×	×	×
Cys	√	√	√	×	×	×	×	×
Met	√	√	√	×	×	×	×	×
Lys	√	√	√	×	×	×	×	×
Gly	√	√	√	×	×	×	×	×
1	√	√	√	√	×	×	×	×
2	√	√	√	√	×	×	×	×
3	√	√	√	√	×	×	×	×
4	√	√	√	√	×	×	×	×
5	√	√	√	×	×	×	×	×
6	√	L-	×	×	×	×	×	×
7	√	√	√	√	√	√	×	×

[b] √: stable gel, ×: gel collapse, L/D-: enantiomeric collapse

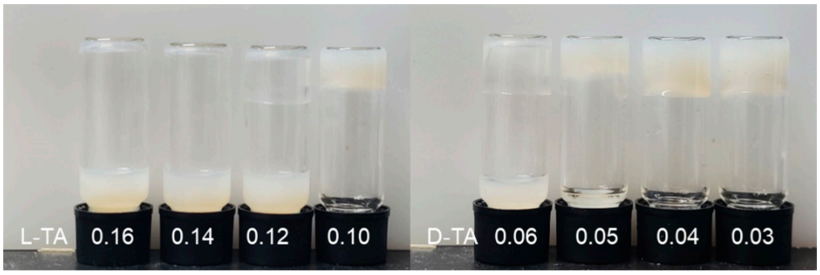

Figure S10. (*S*)-H_6L-$Cu_{0.2}$ gel in the presence of L-TA (0.16 eq., 0.14 eq., 0.12 eq., 0.10 eq.) and D-TA (0.06 eq., 0.05 eq., 0.04 eq., 0.03 eq.) with different equivalents.

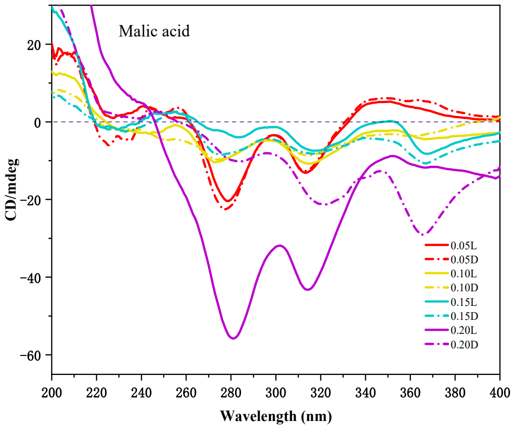

Figure S11. CD spectra of (*R*)-H_6L-$Cu_{0.2}$ gel (4.5 mM) with the addition of Malic acid in EtOH/H_2O (1/1, *v/v*).

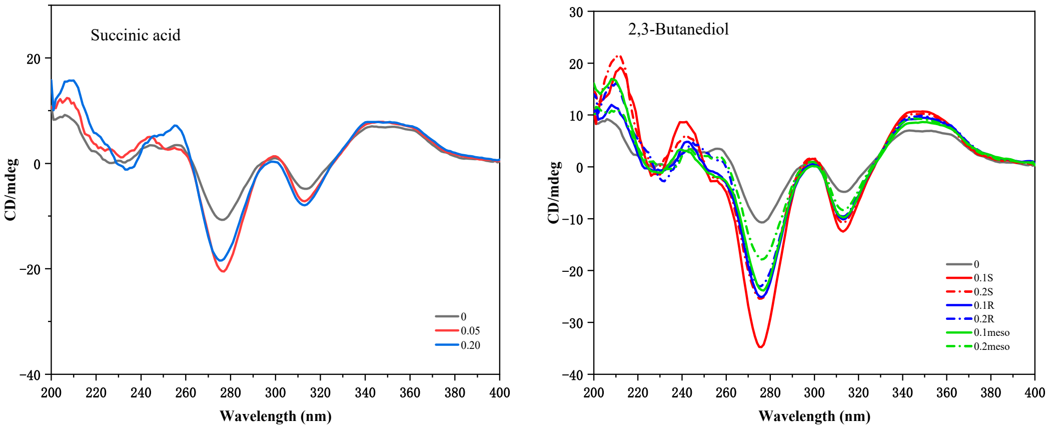

Figure S12. CD spectra of (*R*)-H_6L-$Cu_{0.2}$ gel (4.5 mM) with the addition of Succinic acid and 2,3-Butanediol in EtOH/H_2O (1/1, *v/v*).

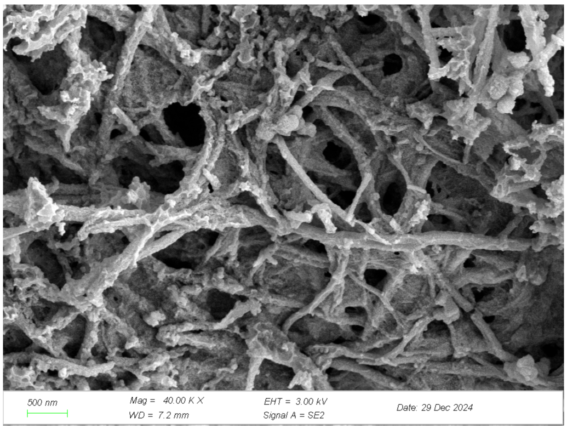

Figure S13. SEM image of (*R*)-H_6L-$Cu_{0.1}$ (4.5mM).

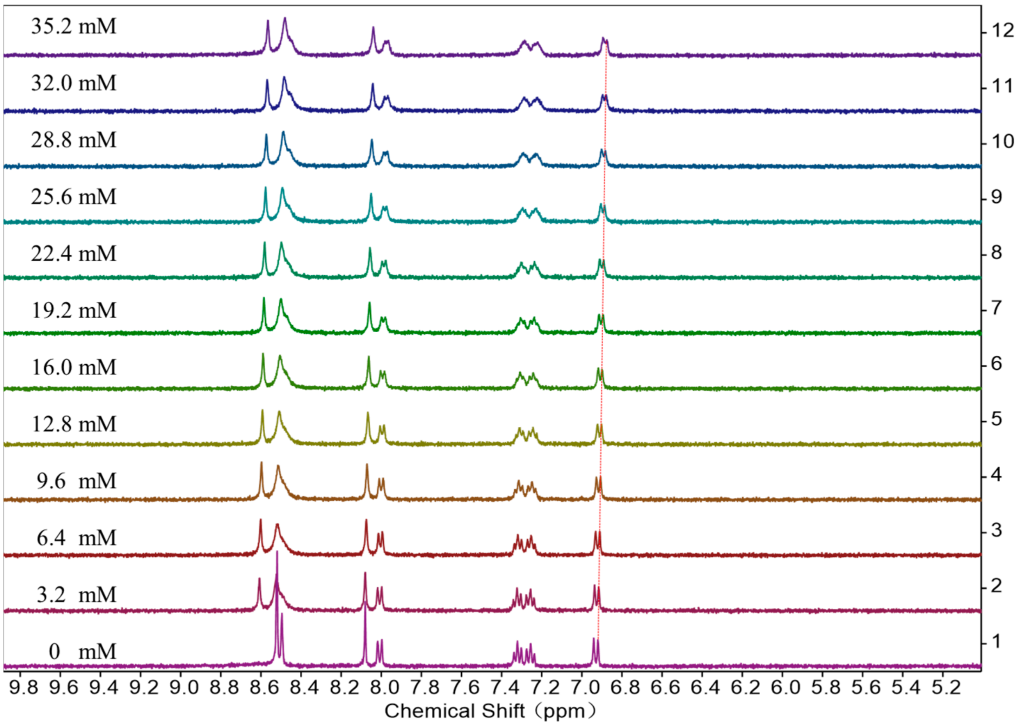

Figure S14. ^1H NMR (400 MHz, 298K) of 16 mM (*R*)-H_6L titrated with Cu^{2+} in DMSO-d_6.

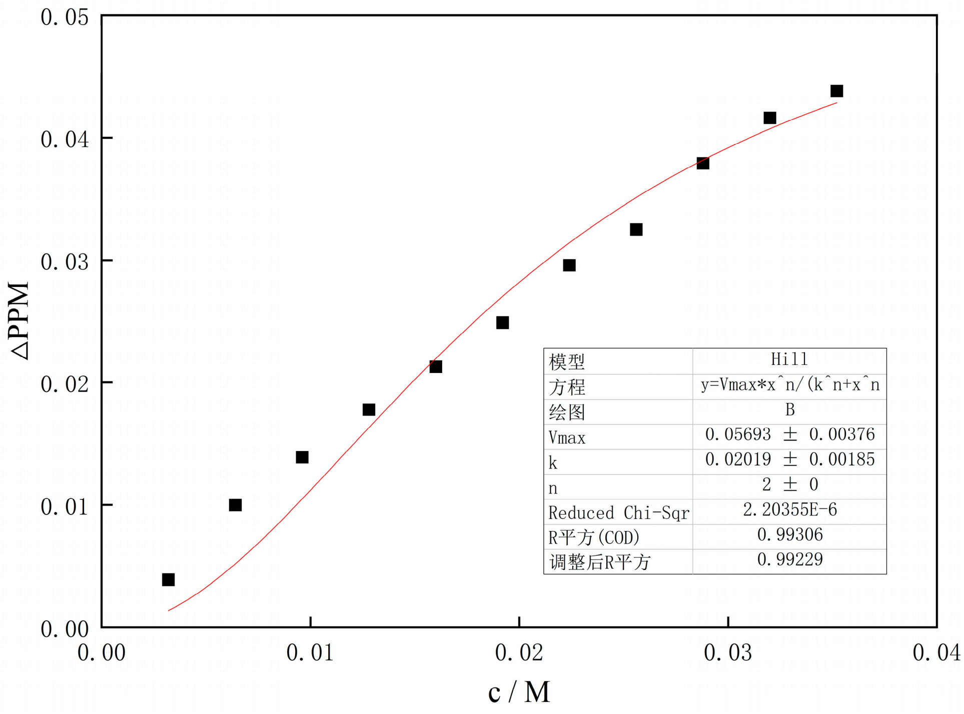

Figure S15. Determination of host-guest binding constants by analyzing proton H^1 shifts in ^1H NMR titration. The binding constants of (*R*)-H_6L with Cu^{2+} was 2.45×10^3 M^{-2}.
